# Coded Aperture Hyperspectral Image Reconstruction

**DOI:** 10.3390/s21196551

**Published:** 2021-09-30

**Authors:** Ignacio García-Sánchez, Óscar Fresnedo, José P. González-Coma, Luis Castedo

**Affiliations:** 1 Department of Computer Engineering & CITIC Research Center, University of A Coruña, Campus de Elviña s/n, 15071 A Coruña, Spain; oscar.fresnedo@udc.es (Ó.F.); luis@udc.es (L.C.); 2 Defense University Center, The Spanish Naval Academy, University of Vigo, Plaza de España 2, Marín, 36920 Pontevedra, Spain; jose.gcoma@cud.uvigo.es

**Keywords:** compressive sensing, hyperspectral imaging, CASSI, sparse estimation algorithms, snapshot devices, system evaluation

## Abstract

In this work, we study and analyze the reconstruction of hyperspectral images that are sampled with a CASSI device. The sensing procedure was modeled with the help of the CS theory, which enabled efficient mechanisms for the reconstruction of the hyperspectral images from their compressive measurements. In particular, we considered and compared four different type of estimation algorithms: OMP, GPSR, LASSO, and IST. Furthermore, the large dimensions of hyperspectral images required the implementation of a practical block CASSI model to reconstruct the images with an acceptable delay and affordable computational cost. In order to consider the particularities of the block model and the dispersive effects in the CASSI-like sensing procedure, the problem was reformulated, as well as the construction of the variables involved. For this practical CASSI setup, we evaluated the performance of the overall system by considering the aforementioned algorithms and the different factors that impacted the reconstruction procedure. Finally, the obtained results were analyzed and discussed from a practical perspective.

## 1. Introduction

Spectral imaging refers to techniques focused on capturing images from various wavelength bands, thus providing spatial and spectral information of scenes. For instance, RGB images contain information from bands within the visible region of light. In contrast, spectral imaging covers a wider spectrum range including nonvisible light. Hyperspectral imaging is usually mixed with multispectral imaging, but even though both are spectral imaging techniques, they differ in that the first considers a high number of continuous spectral bands, while multispectral imaging deals with a lower amount of discrete wider bands.

In general, spectral data are valuable as they allow extracting relevant information from scenes by considering their spectral properties. This has been shown to have great potential to be applied in numerous fields such as medicine [1,2], astronomy [3], agriculture [4,5], or surveillance [6]. Nevertheless, despite being a solid and active field of study, there still exist some limitations that prevent the popularization of the new techniques emerging from hyperspectral technology:

- Hyperspectral imagers are quite expensive even to this day;
- There is a lack of accessible hyperspectral image repositories;
- Processing algorithms designed as extensions of the ones used for processing RGB images could potentially miss certain special characteristics inherent to the spectral properties of the data.

Numerous hyperspectral imagers have arisen in the last few years. Their focus is to acquire a three-dimensional datacube from a scene, with two spatial and one spectral dimension. Datacubes can be encapsulated in four different ways depending on how the data are captured [7]:

- Whiskbroom: A point of information is captured over the whole spectral information at once [8]. Scanning point-by-point both spatial dimensions and mapping each capture to a 2D detector would yield a complete datacube;
- Pushbroom: A slit image of the scene containing spatial information along one axis and spectral information along the other is obtained at once [9]. Each slit image is mapped onto a 2D detector. Scanning the spatial dimension perpendicular to the one of the slit images provides a complete datacube;
- Staring: A single spectral band is captured at once containing the full spatial information over a certain spectral slice. Changing the output wavelength of the filter over time leads to a complete datacube;
- Snapshot: The three dimensions of the information are captured in a single shot with the help of a dispersive element, which allows sensing the different wavelengths on the spectrum at once [10,11,12]. Then, mapping the data onto a 2D detector leads to a complete datacube.

In this work, we focused on the use of a snapshot imager to acquire the hyperspectral data. More specifically, the CASSI method was considered as the strategy for sensing the hyperspectral information of the scenes [13,14,15].

An important issue when dealing with hyperspectral images is the fact that having a third (spectral) dimension largely increases the size of the images. In this way, depending on the systems using this technology, memory and storage requirements may be a problem. For instance, IoT devices such as drones, which are typically used for these types of applications, could potentially have problems dealing with the processing or even the transmission of hyperspectral data. For this reason, the use of schemes based on CS is fundamental.

Traditional approaches to compression are oriented toward sampling based on the Nyquist criterion, which states the minimum number of samples required to correctly recover some particular data. In contrast, CS goes beyond the Nyquist limits and allows recovering an image using a reduced amount of samples. As a result, huge compression rates can be achieved while recovering an acceptable approximation to the original data [16]. This is possible because CS exploits certain properties and redundancies from compressible measurements of the original data [17,18,19]. Nonetheless, this comes at the cost of a much more complex process and the need to incorporate the data properties to define adequate optimization problems.

These problems are partially solved by the multiple existing algorithms in the literature that obtain accurate reconstructions of the input data from their compressed measurements [20,21]. Some of the most relevant ones are OMP [22,23], which belongs to the greedy iterative methods, GPSR [24], which is a type of projected gradient algorithm, LASSO [23], whose formulation is based on a convex relaxation of the original estimation problem, IST [25] and Tw-IST [26] as representative methods of the iterative thresholding techniques, and the AMP [27,28] algorithm based on message-passing approaches, among many others.

The use of a snapshot imager based on CASSI allows integrating the principles of CS theory in a simple way [14]. The whole sensing procedure can be modeled from the CS perspective, leading to a mathematical formulation that fits the general sparse estimation problem. However, the large size of hyperspectral images can complicate the reconstruction procedure if it is directly applied to the entire datacube. For that, it is essential to carry out a partial reconstruction process that ensures an affordable computational cost [29]. In this case, the information obtained after the sensing step is split into nonoverlapping blocks, which are employed to obtain partial reconstructions of the original data. Then, such partial reconstructions have to be assembled to generate an estimation of the image datacube. This blocking-based approach considerably complicates the CASSI model due to the dispersion effect in the sensing process of the snapshot imager. Furthermore, depending on the specific application for which these images are taken, quality could be a trade-off for faster processing and vice versa. This is the case of some real-time applications such as surveillance, for instance. Some parameters such as the size of each partial reconstruction or the number of measurement shots could impact these factors, and the model settings have to be adapted for each particular application. For this reason, it is important to analyze the performance of the different reconstruction algorithms and the impact of the parameters involved in the design of practical CASSI systems.

In this paper, we aimed at implementing and evaluating a functional block model based on the CASSI scheme that was proposed in [29] in the context of hyperspectral images sampled with CASSI. For this setup, we profusely describe the CS formulation required for the modeling of the sensing and reconstruction procedures. We also provide the sequence of steps for the construction of the main variables, especially the measurement matrix, considering the physical particularities of the CASSI sensing and the corresponding block structure. From the resulting system implementation, we analyzed the different parameters that impact the performance of the reconstruction of the hyperspectral images and measure such impact according to different evaluation metrics. Hence, the main contributions of this work can be summarized as:

- A comprehensive overview of the block CASSI model is presented with all the details required for the implementation of a practical sensing and reconstruction system based on this technology;
- A comparison between several traditional reconstruction sparse algorithms is made for the considered scenario;
- A detailed analysis of the different parameters that influence the reconstruction of hyperspectral images is carried out. It is supported by a large variety of computer experiments in which the obtained results are included and discussed.

The rest of the paper is organized as follows. Section 2 explains the fundamentals for the sensing procedure of hyperspectral images with CASSI. The modeling of the CASSI-based sensing according to the CS framework and the corresponding reconstruction/estimation procedure are described in Section 3. Section 4 presents the block CASSI model to enable a practical reconstruction of the hyperspectral images. Section 5 describes the computer experiments carried out in this work, and the obtained results are shown for each experiment. Finally, a discussion of the most relevant points found in this study is conducted in Section 6, and Section 7 is devoted to the general conclusions of the work.

## 2. Hyperspectral Images Sensing

As discussed, hyperspectral images are composed by a large number of spectral bands for a given range of wavelengths. Therefore, their data are represented by a 3D matrix, called a datacube, which is composed of data in two spatial dimensions and a spectral one. In particular, each spatial position in a datacube takes several values across the spectral dimension for the same pixel. It is worth mentioning that these images, considering the CS framework, are represented by some vectorized measurements that the hyperspectral camera senses.

In this work, the vectorized measurements from the hyperspectral images were obtained from a prototype hyperspectral camera whose hardware components form a setup referred to as CASSI. This setup was specifically developed to take advantage of the principles of CS for hyperspectral imaging. As the design of the measurement matrix and the entire reconstruction procedure directly depend on this setup, we briefly describe the basis for the generation of the image measurements by means of CASSI.

**Figure 1:**
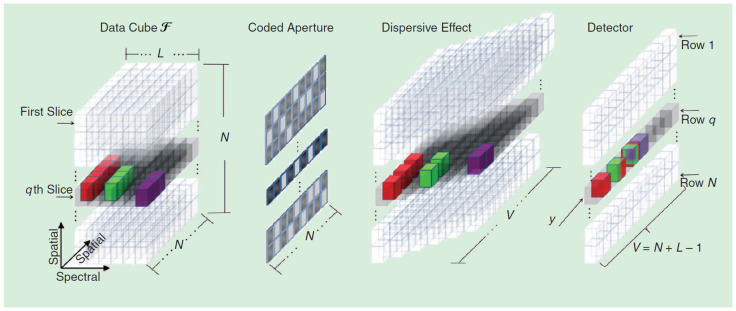
Spectral optical flow in CASSI, reprinted from ref. [14] Copyright 2013 IEEE.

As observed in Figure 1, four main components should be considered in the procedure to obtain the CASSI measurements from a hyperspectral image:

- Image data: In hyperspectral imaging, this information is represented by a datacube (various spectral slices over two spatial dimensions). These data hence correspond to the discrete representation of the scene captured with the CASSI camera across the considered spectral bands. Let us denote $f_{0}\left(x,y,\lambda \right)$ as the input information from the scene, where (x,y) are the spatial coordinates and λ represents the wavelength for a particular spectral band. The corresponding discrete datacube is modeled by a 3D matrix, F(x,y,z), with dimensions N×N×L, where *N* determines the spatial resolution and *L* the number of spectral bands;
- Coded aperture: Grids are used to block or unblock the wavelengths of electromagnetic radiation at each spatial coordinate by following a known coded pattern in order to cast a “shadow” upon a plane, which in this case would be the radiation detected by the camera sensor. Their main goal is to obtain data samples with a structure that allows compression while undersampling the data in a way in which measurements obtained from them are highly compressed despite retaining sufficient information to obtain an accurate reconstruction. Let us denote $T\left(x,y\right)\in F_{2}^{N\times N}$ as the binary matrix that represents the coded aperture pattern applied to the spectral information of the scene;
- Prism: This acts as a dispersive element shifting each of the spectral slices along the columns of the datacube. In practice, this means that each 2D matrix representing a fixed wavelength will be moved one column to the right from the previous;
- FPA detector: This corresponds to the image sensing element consisting of an array (typically rectangular) of light-sensing pixels at the focal plane of a lens. In this sensing step, the dispersed information corresponding to the different spectral bands is hence integrated into a single 2D matrix. This resulting matrix will contain the compressed representation of the hyperspectral images and will be called a measurement shot or, directly, a shot.

Considering these components in the CASSI setup, we next summarize the procedure to obtain the compressive measurements:

1. An optimized coding was first applied to the spectral information of the scene, i.e., $f_{0}\left(x;y;\lambda \right)$. It is worth highlighting that the same aperture pattern given by T(x,y) was applied to all the *L* spectral bands obtained with the CASSI camera to generate a measurement shot. Thus, the same spatial coordinates were blocked or unblocked across the spatial dimension. However, note that different aperture patterns would be used for different measurement shots in order to obtain different compressive representations;
2. The result from applying these coded apertures is $f_{1}\left(x;y;\lambda \right)$, which was subsequently modified by the dispersive element (prism) as described above, thus producing the dispersed data $f_{2}\left(x;y;\lambda \right)$. It is important to note that the coded aperture pattern and the dispersive effect would decisively determine the construction of the measurement matrix in the general formulation of the CS problem;
3. Finally, these dispersed data were sensed by the FPA detector and integrated into a two-dimensional FPA measurement shot. In this way, the dispersed information from the different spectral bands at the same spatial coordinate collapsed into the same area of the FPA detector, and such information was hence combined to obtain a single value into the corresponding entry of the matrix that defines the measurement shot. Let us denote $Y\left(x,y\right)\in R^{N\times V}$ as the matrix that contains the compressive measurements of the hyperspectral image. Note that the number of columns of the matrix *Y* is given by V=N+L−1 due to the aforementioned dispersive effect of the prism.

In the ensuing section, we explain the mathematical formulation modeling the generation of the measurement shots from an input datacube according to the CS theory.

## 3. CS Formulation

Sparsity is a fundamental property for the successful application of CS techniques. This framework can be used to acquire and reconstruct signals or images from a limited number of linear projection measurements at sub-Nyquist sampling rates [16]. In particular, a signal is sparse in some domain if a large portion of its values in such a domain are zeros. However, images are not generally sparse in the natural domain as most pixels contain values different from zero. However, the use of domain transforms allowed us to obtain a sparse representation of an image in a different domain, thus making it suitable for the application of the tools provided by CS. In particular, linear discrete transforms such as the DCT or the DWT can be used to obtain a sparse representation of the hyperspectral images [30].

Mathematically, any linear discrete transform can be represented as:

(1) x=Ψs,

where $x\in R^{N}$ is the vector that contains the coefficients in the new domain, $\Psi \in R^{N\times N}$ is the matrix that comprises the vectors of the basis, and $s\in R^{N}$ represents the data vector in the original domain, which was assumed to be K-sparse (i.e., the number of nonzero elements in x is K). The basis matrix Ψ was assumed to be unitary (i.e., $\Psi \Psi^{T}=I_{N}$ where $I_{N}$ is the identity matrix of dimension *N*), and thus, the vector s in the original domain can be recovered as:

(2) $$s=\Psi^{T}x,$$

Assuming that x is sparse in the domain defined by the basis in Ψ, we can formally model the operation of CS-based measurement as:

(3) $$\begin{array}{c} y=\Phi s=\Phi \Psi^{T}x=Ax, \end{array}$$

where $s\in R^{N}$ is the input data, $y\in R^{M}$ is the vector that contains the *M* projection measurements of the input data, $\Phi \in R^{M\times N}$ is the random projection matrix, and $A=\Phi \Psi^{T}\in R^{M\times N}$ is the overall CS matrix, referred to as the measurement matrix. In this case, the original data would be represented by the *M* compressive measurements in y with M≪N.

An important issue in the CS framework is the number of measurements *M* required to accurately recover the input data from their compressive measurements. This is closely related to the properties of the projection matrix Φ. In particular, the choice of an incoherent dictionary for the CS projection ensures a unique identification of sparse data from a minimum number of measurements [31,32]. In general, the suitability of the projection matrix can be measured from its mutual coherence as:

(4) $$\mu \left(\Phi \right)=\max_{i\neq j}\;\left|\langle \phi_{i},\phi_{j}\rangle \right|,$$

i.e., as the largest absolute inner product between different columns of the matrix Φ. For the particular case of applying CS in a transformed domain (as in this work), the fundamental aspect for the design of the measurement projections is the mutual coherence between the basis matrix Ψ and the resulting projection matrix Φ [17], which can be defined as:

(5) $$\mu \left(\Psi ,\Phi \right)=\max_{i,j}\;\left|\langle \psi_{i},\phi_{j}\rangle \right|,$$

i.e., as the largest absolute inner product between any two columns of the matrices Ψ and Φ. Several works have addressed the design of the projection matrix considering both random projections of the sparse data [17,18,33,34] or more structured dictionaries [35,36,37,38,39].

### 3.1. Sparse Estimation from Compressive Measurements

Once the CS measurements are generated and the measurement matrix is properly defined, the data are ready to be stored or transmitted efficiently. At this point, the reconstruction of the original data from the compressive measurements requires solving a sparse estimation problem. In general, this problem can be formulated as:

(6) $$\mathrm{find}\;\;\hat{x}\;\mathrm{subject}\mathrm{to}\;\;A\hat{x}\approx y\;\;\;\mathrm{and}\;\;\;\hat{x}\;\mathrm{is}\mathrm{sparse},$$

where y is the vector corresponding to the measurements and A is the same measurement matrix used in the sampling step. It is worth remarking that the equation system $A\hat{x}=y$ is indeterminate, and therefore, the optimization problem in Equation (6) aims at forcing solutions with a low number of nonzero coefficients. Furthermore, the formulation above focuses on recovering the data in the sparse domain, and therefore, we need to carry out the additional step $\hat{s}=\Psi^{T}\hat{x}$ to recover the input data in the original domain.

Classical techniques to solve linear inverse problems generally produce nonsparse solutions. Hence, it is required to develop specific algorithms that are able to provide sparse solutions for the estimation problem in Equation (6). Different algorithms have been proposed in the literature depending on the approaches considered to deal with the intrinsic sparsity constraint in the sparse estimation problem. A natural approach consists of introducing the $\ell_{0}$ norm in the optimization problem to guarantee sparse solutions, i.e.,

(7) $$\begin{array}{c} \min_{x}\;{∥x∥}_{0}\;\mathrm{subject}\mathrm{to}\;{∥\mathrm{Ax}-y∥}_{2}<\epsilon , \end{array}$$

where ϵ≥0 is the error tolerance and the operator ${∥\;\cdot \;∥}_{p}$ represents the $\ell_{p}$ norm. According to the problem above, we actually aim at balancing two objectives:

- Large sparsity levels for the solution x by using the $\ell_{0}$ norm;
- Low difference between y and Ax by using the $\ell_{2}$ norm.

Using a mixed formulation, we can introduce a regularization parameter τ>0 to help in balancing the previous conditions such that:

(8) $$\min_{x}\;\frac{1}{2}{∥Ax-y∥}_{2}^{2}\;+\;\tau {∥x∥}_{0}.$$

As observed, the parameter τ defines a trade-off between the two objectives for the solutions of the sparse estimation problem. Small values for this parameter prioritize the left term of the cost function in (8), i.e., to minimize the difference between the estimation and the actual solution, while large values lead to sparser solutions.

However, by containing the $\ell_{0}$ norm, the cost function in (8) is not convex, and it necessarily leads to a combinatorial problem. Different alternatives have been proposed to deal with this issue, including greedy approaches such as pursuit methods, convex relaxation of the natural problem, or Bayesian methods. In the following, we provide a brief overview about some of the most common algorithms proposed to solve the sparse estimation problem that were considered in this work:

- OMP: This is an effective approach and one of the simplest algorithms that forms a part of the greedy pursuit approaches. OMP defines a refinement iterative algorithm where the best candidate from a dictionary is chosen at each step. The dictionary comprises the column vectors from the measurement matrix A, and therefore, the best candidate corresponds to the vector of the dictionary that most contributes to decreasing the difference between the current solution and the actual one (see the left term in (8)). After selecting the best candidate, the solution vector is updated, and the iterative algorithm continues the refinement procedure to minimize the residual term ${∥\mathrm{Ax}-y∥}_{2}^{2}$.
  Greedy pursuit approaches generally provide near-optimal sparse approximations for incoherent dictionaries [27]. This premise can be extended for our scenario as long as the dictionary elements and the basis of the sparse domain also satisfy mutual incoherence. The main issue of the basic version of OMP is related to its computational complexity because the number of required iterations is given by the number of nonzero components of the vector to be reconstructed. Hence, its computational cost is acceptable for very sparse solutions, but it could be large otherwise. Related to this issue, it can lead to inaccurate estimations for input data with a large amount of small values in the sparse domain.
  To solve these drawbacks, more sophisticated methods have been developed based on selecting multiple columns per iteration or pruning the set of active columns at each step (see [23] and the references therein). In this work, we focused on the basic version of OMP because the hyperspectral images were expected to be sufficiently sparse in the frequency domain:
- LASSO: Another fundamental approach for sparse reconstruction consists of employing a convex relaxation of the natural sparse estimation problem. This is performed by replacing the nonconvex $\ell_{0}$ norm in (8) by the $\ell_{1}$ norm, which is the closest convex function to $\ell_{0}$. Therefore, the optimization problem following this strategy reads as:
  (9) $$\min_{x}\;\frac{1}{2}{∥Ax-y∥}_{2}^{2}\;+\;\tau {∥x∥}_{1}\;,$$
  and the cost function is now convex. In this case, the cost function includes the quadratic error term combined with a sparseness-inducing ($\ell_{1}$) regularization term. As a side note, the $\ell_{1}$ minimization techniques can be optimized by using *interior-point* methods, first-order *gradient projection* methods, or *homotopy* methods, although the latter only works properly for small-scale problems [40].
  It is also worth noting the difficulty in selecting an appropriate value for τ in advance. Therefore, we would actually need to solve the problem above repeatedly for different choices of this parameter and track the obtained solutions. An equivalent problem to (9) is given by the LASSO formulation, which is stated as:
  (10) $$\min_{x}{∥Ax-y∥}_{2}^{2}\;\;\mathrm{subject}\mathrm{to}\;{∥x∥}_{1}\leq \beta \;,$$
  with β a positive parameter, which plays a similar role to the regularization parameter.
  There exists several works that have analyzed the convergence of LASSO for the estimation of sparse signals [31,41,42]. Such works confirmed the suitability of LASSO-formulation-based algorithms as they provide accurate approximations for incoherent dictionaries even for noisy CS measurements [41,43].
  Although OMP and LASSO solve different minimization problems, they are based on the same principles. Both approaches start from an all-zero solution and, then, iteratively construct a sparse solution by considering the correlation between columns of the measurement matrix A and the current residual vector. However, OMP usually requires fewer iterations than LASSO to converge to the final solution due to the strategy of handling the active set of candidates. On the contrary, OMP produces less accurate solutions when there is a certain correlation between the dictionary elements (columns of A);
- GPSR: This is one of the *gradient projection* methods. These iterative algorithms solve the relaxed convex problem in (9) by updating the solutions at each step with the gradient of the cost function. These kinds of algorithms have been demonstrated to perform well in a wide range of applications, although they tend to degrade as the regularization term is de-emphasized [24]. Indeed, gradient-based methods are especially suitable for the estimation of very sparse signals where the weight of the regularization terms is significant. Their main advantage is that they are considerably faster than other methods in spite of the fact that they also require solving a set of problems for different values of the regularization parameter. GPSR is able to efficiently solve a sequence of problems such as (9) for a sequence of values of τ.
  There exist two main approaches for the implementation of GPSR-based algorithms, although both share the same underlying formulation. The first approach is GPSR-Basic [24], which is similar to a step descent approach. The optimization problem is hence reformulated according to a BCQP formulation such that the resulting cost function is minimized following the negative gradient direction. The iterative solutions are then projected onto the feasible set of solutions determined by the problem constraints. A backtracking line search is also performed to optimize the step parameter.
  The second approach is the Gradient Projection Barzilai-Borwein (GPSR-BB) algorithm [44]. The main difference with respect to the previous approach is the choice of the step size, which is based on an approximation for the Hessian of the cost function. As a consequence of this choice, the objective function does not necessarily decrease at every iteration, unlike the basic version of GPSR. However, this particular choice for the gradient algorithm step has been proven analytically to produce accurate solutions for simple problems [44];
- IST: Iterative thresholding algorithms [45] are some of the simplest known techniques for sparse reconstruction, along with greedy approaches. Typical iterative thresholding algorithms update the solution at each iteration as follows:
  (11) $$\begin{array}{c} x_{\left[n+1\right]}=\Lambda_{\alpha }\left(x_{\left[n\right]}+A^{T}\left(y-Ax_{\left[n\right]}\right)\right), \end{array}$$
  where $\Lambda_{\alpha }\left(\cdot \right)$ represents the thresholding function, which is applied elementwise to obtain a sparse solution and α is the threshold considered in $\Lambda_{\alpha }\left(\cdot \right)$. These algorithms start with an initial solution $x_{\left[0\right]}=0$. As observed, the update rule in (11) decomposes the sparse estimation into two separate steps: the unconstrained minimization of the objective function and the thresholding operation to satisfy the sparsity constraint. Hence, these iterative algorithms are able to converge to a local minimum of the optimization problem in (8) under certain conditions [45]. In general, these algorithms have empirically been shown to provide good sparse estimations if enough sparsity is present at the input data and an appropriate threshold is chosen.
  Iterative thresholding algorithms are mainly classified into two categories depending on the type of considered thresholding operator: IHT and Iterative Soft Thresholding (IST). In this work, we focused on IST as it generally provides better results for sparse reconstruction. The algorithms based on IST are especially suitable for well-conditioned problems since they have been shown to present slow convergence when the sensing matrix is ill-conditioned. An alternative to solve this issue is the use of the Tw-IST approach, which was proposed in [26] to accelerate the convergence rate of IST algorithms with this class of problems. The main difference of the Tw-IST algorithm with respect to the basic versions of IST is to consider the two previous estimates at each iterate to update the current estimation, rather than considering only the previous one. This modification in the update step has been shown to provide significant gains in terms of execution time for ill-conditioned problems and moderate ones otherwise.

All these algorithms address the sparse estimation problem from a similar perspective, but they present clear algorithmic differences that make them suitable for different situations and different purposes. OMP is an adequate approach for input data with a large sparsity level, and it imposes severe restrictions on the dictionary matrix to perform successfully. LASSO is more flexible and generally provides accurate estimates, but its complexity is usually superior. The GPSR family of algorithms stand out for their lower computational cost and smaller required execution time. They are generic algorithms that can be suitable for a large range of applications, including scenarios with a lower sparsity level. Their performance, however, is usually worse than the previous strategies for sparse data. Finally, IST constitutes a trade-off between performance and computational cost, but the choice of an appropriate threshold parameter is critical. In this case, Tw-IST could also be employed to improve the convergence rate of the estimation algorithm and, consequently, the time required for reconstruction, in the case of having ill-conditioned measurement matrices.

For these reasons, the selection of the reconstruction strategy for CS-based applications is not trivial and depends on multiple factors. The analysis of the reconstruction of hyperspectral images from the CASSI measurements is hence a key point to evaluate the practical suitability of the CASSI setup for sampling hyperspectral images.

### 3.2. CS Formulation for the CASSI Setup

As introduced, the CASSI model fits with the general formulation of any CS problem. The input datacube corresponding to the hyperspectral image is represented by a smaller set of compressive measurements, which are obtained following the procedure described in Section 2. These measurements are then considered to obtain an accurate reconstruction of the original hyperspectral image by using some CS estimation algorithm.

In particular, the CASSI-based sensing produces a 2D matrix of N×V FPA measurements (shot) from the original N×N×L datacube. Recall that *N* represents the spatial dimensions in the original datacube and V=N+L−1 is the dimension of the spectral dispersive effect, which depends on the number of spectral bands *L*. By stacking the values of the 3D datacube into a single vector, the CS formulation for the CASSI setup is:

(12) $$y=Hf=H\Psi^{T}x=Ax,$$

where $f\in R^{N^{2}L\times 1}$ is the vector containing all the elements of the 3D datacube F, $y\in R^{NV\times 1}$ is the vector of FPA measurements, and $A=H\Psi^{T}$ is the CASSI measurement matrix enclosing $H\in R^{NV\times N^{2}L}$, which accounts for the effects of the coded aperture and the dispersive effect of the prism. Furthermore, we have $\Psi \in R^{N^{2}L\times N^{2}L}$, which represents the basis for the domain in which f is sparse. As observed, this CS formulation is equivalent to the general formulation of the CS problem in (3) with M=NV and H playing the role of the random projection matrix Φ.

The formulation in (12) requires rearranging the 3D datacube in a vector form. In this case, the basis matrix Ψ should be able to provide a sparse representation of the input data through the three existing dimensions (the two spatial and the spectral one). As presented in [46], an appropriate mechanism to generate a sparsifying basis for multidimensional CS scenarios is by means of the Kronecker product. Hence, a basis matrix is selected to provide a sparse representation per dimension, and the overall basis matrix is obtained as the Kronecker product of the individual bases. We obtained a single sparsifying basis for the whole hyperspectral image as the Kronecker product of the sparsifying bases of each of the dimensions, i.e.,

(13) $$\Psi =\Psi_{1}⊗\Psi_{2}⊗\Psi_{3},$$

where $\Psi_{1}\in R^{N\times N}$ and $\Psi_{2}\in R^{N\times N}$ correspond to the two spatial dimensions and $\Psi_{3}\in R^{L\times L}$ is the basis for the spectral one. The usual choices to obtain a sparse representation of natural images are the wavelet transform or the DCT [47,48]. The former bases are known to be mutually incoherent with structured projection matrices or even random projections for the sensing step [36,37,49]. The DCT is a transform widely employed in image compression and also in the context of CS [50]. The DCT is especially interesting for the considered scenario because it is able to efficiently compact the signal energy when the input is highly correlated and using a relatively small block size [48].

**Figure 2:**
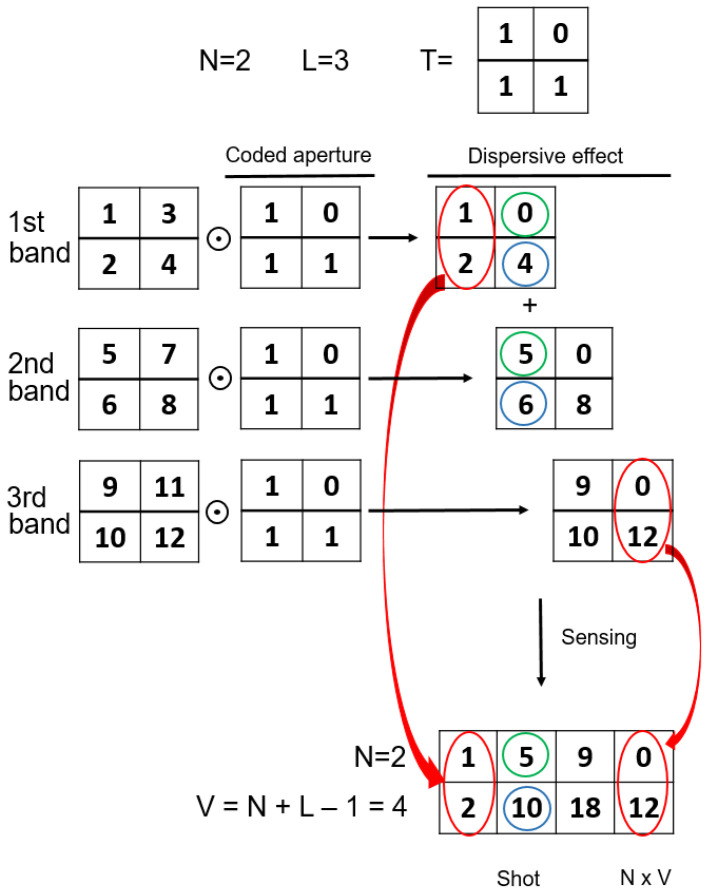
A simple example of the CASSI sensing procedure with N=2 and L=3.

**Figure 3:**
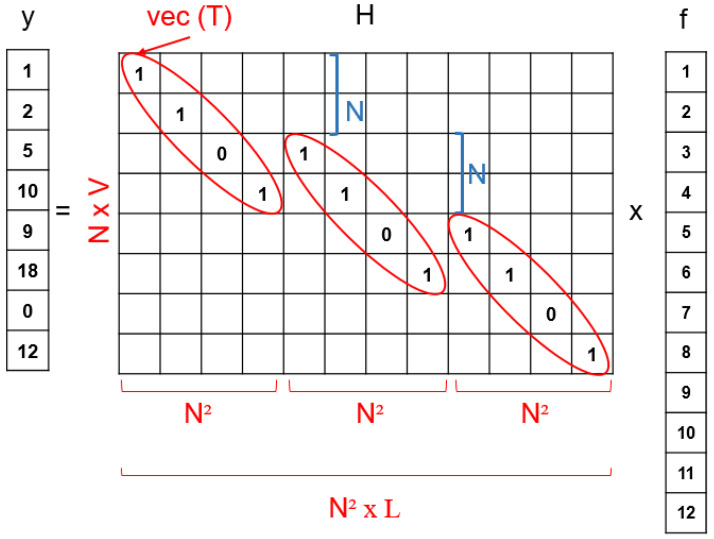
Equivalent CS formulation with the particular structure of the projection matrix H.

At this point, one of the essential issues is the construction of the projection matrix H. This matrix should incorporate the three main steps of the CASSI measurement procedure explained in Section 2: (1) coded aperture, (2) dispersive effect; (3) FPA sensing. Figure 2 and Figure 3 show a small example of the CASSI-based sensing procedure and the corresponding equivalent CS formulation in (12), including the ideas for a systematic construction of the matrix H from the matrix T, which specifies the coded aperture pattern. As observed, we start from a simple datacube with L=3 spectral bands and 2×2 spatial dimensions, i.e., N=2. These spectral slices are individually coded with the matrix T and dispersed by the prism effect (in practice, they are shifted one column to the right). Finally, the values at the same spatial coordinates are summed to simulate the sensing in the pixels of the FPA sensor. According to this procedure, the matrix H can be constructed as shown in Figure 3 by considering the equivalent vector CS formulation. The vector representation of the matrix T is placed at the matrix H following the same pattern:

- We first place the entries of T at the main diagonal;
- We next move $N^{2}$ positions in the horizontal dimension and *N* positions in the vertical one and place the entries of T again;
- We repeat this procedure *L* times to complete the construction of the matrix H;
- The rest of elements are set to zero.

As introduced at the beginning of the section, the key point to obtain an accurate reconstruction of the original information from a minimum number of compressive measurements is to guarantee the mutual incoherence between the columns of the sparsifying basis matrix Ψ and the columns of the projection matrix H. As observed in Figure 3, the matrix H has a particular structure, which eventually leads to selecting one or several columns of the basis matrix Ψ to determine the overall sensing matrix A. This particular construction of H hence ensures that the elements of the dictionary used for the sensing of the hyperspectral images are incoherent, and as consequence, they should produce an appropriate set of compressive measurements.

The CS formulation in (12) can be extended to the case of having several measurement shots from the input hyperspectral image. In particular, we have:

(14) $$y_{i}=H_{i}f=H_{i}\Psi^{T}x=A_{i}x,\;\;i=1,\ldots ,N_{s}$$

where the subindex *i* refers to the *i*-th shot and $N_{s}$ indicates the number of available shots. As observed, the measurement matrix A depends on the specific shot since a different coded aperture pattern is used for each shot. The equation above can be expressed in a more compact way as follows:

(15) $$y_{v}=H_{v}\Psi^{T}x,$$

where $y_{v}={\left[y_{1}^{T},\ldots ,y_{N_{s}}^{T}\right]}^{T}$ and $H_{v}={\left[H_{1}^{T},\ldots ,H_{N_{s}}^{T}\right]}^{T}$. Therefore, we can individually construct each projection matrix $H_{i}$ following the procedure explained above and obtain $H_{v}$ by stacking the resulting matrices vertically.

Note that this formulation still matches the general CS problem in such a way that we can use the traditional approaches for the reconstruction of the hyperspectral image from the vector of compressed measurements $y_{v}$. The objective is hence to obtain an accurate and sparse estimate of the vector x from the available shots by using any of the explained algorithms. Then, the 3D datacube corresponding to the input hyperspectral image can be reconstructed from such a sparse estimation with the help of the matrix $\Psi^{T}$.

Unfortunately, the computational complexity of the reconstruction procedure increases as the resolution of the images becomes larger (the dimension of vector x quadratically grows with *N*). However, we can circumvent this issue by using an alternative approach known as the block CASSI model where the overall problem is divided into a set of smaller-scale subproblems [29]. This allows us to reconstruct the underlying datacube by using a set of reconstructions obtained from nonoverlapping FPA windowed shots, as we explain in the ensuing section. In addition, each block can be reconstructed independently in a parallel fashion.

## 4. Practical Images Reconstruction Based on a Block Model

Exploiting the physical properties of the CASSI optical sensing phenomena, we can reconstruct the underlying 3D datacube from a set of reconstructions obtained from nonoverlapped FPA windowed measurements. This means addressing the reconstruction of the datacube using blocks of size B×B instead of using the entire datacube in a single step. This approach is known as the block model in CASSI, and it clearly helps reduce the computational complexity of the reconstruction procedure as the block size decreases.

For this alternative approach, we have to carry out the following steps:

- Dividing the N×V shot into nonoverlapping blocks of a smaller size (B≪N);
- Solving the sparse estimation problem individually for each measurement block in order to obtain an estimate of the datacube chunk, which produces the considered block of measurements;
- Assembling the entire datacube from the partial reconstructions of the different blocks.

**Figure 4:**
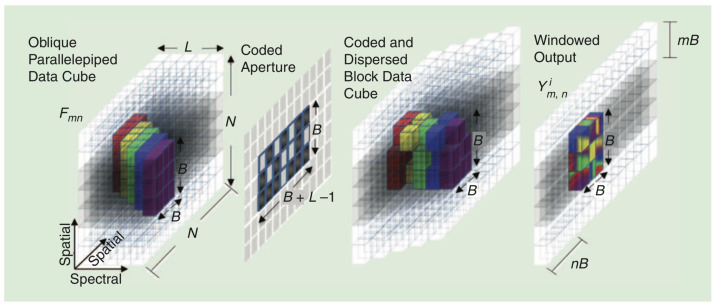
Graphical representation of the impact of using a block model in the CASSI procedure; reprinted from ref. [14] Copyright 2013 IEEE.

It is worth remarking that the B×B portion of the spectral bands, which collapses to the same B×B section at the FPA sector, is previously affected by the dispersive effect of the prism. Thus, the datacube chunk corresponding to a windowed measurement block will actually be a 3D oblique parallelepiped with *L* spectral bands, each one of them shifted one column to the left. Figure 4 illustrates this situation caused by the dispersive effect. As observed, the B×B portion of the *L* bands in the oblique parallelepiped is coded with the corresponding section of the aperture pattern matrix and aligned after going through the prism. Hence, it is fundamental to consider this particular shape of the datacube chunks in order to reassemble the hyperspectral image from the estimates obtained for each B×B measurement block. In addition, it also determines the construction of the partial projection matrices that must be used in each sparse estimation subproblem.

Consider that we have to reformulate the CS problem to accommodate the block CASSI model. The total number of windowed blocks will be $N^{\prime }\times V^{\prime }$ with $N^{\prime }=\left\lceil N/B\right\rceil$ and $V^{\prime }=\left\lceil V/B\right\rceil$. From this, we rearrange the entire *i*-th FPA shot as follows:

(16) $$Y^{\left(i\right)}=\left[\begin{array}{cccccc} Y_{1,1}^{\left(i\right)} & Y_{1,2}^{\left(i\right)} & . & . & . & Y_{1,V^{\prime }}^{\left(i\right)}\\ . & . & . & \; & & .\\ . & . & \; & . & \; & .\\ . & . & \; & & . & .\\ Y_{N^{\prime },1}^{\left(i\right)} & Y_{N^{\prime },2}^{\left(i\right)} & . & . & . & Y_{N^{\prime },V^{\prime }}^{\left(i\right)} \end{array}\right],\;\;\forall i=1,\ldots ,N_{s}.$$

Let $F_{m,n}^{\left(i\right)}$ denote the oblique parallelepiped in the datacube, which determines the measurement block $Y_{m,n}^{\left(i\right)}$. For notational simplicity, we disregard the super-index (i) from now on. In that case, the elements of the matrix above can be obtained as:

(17) $$y_{m,n}=H_{m,n}f_{m,n},\;\;m=1,\ldots ,N^{\prime },\;\;n=1,\ldots ,V^{\prime },$$

where $y_{m,n}\in R^{B^{2}}$ is the vectorized form of $Y_{m,n}$, $H_{m,n}$ is the partial projection matrix corresponding to the current block, and $f_{m,n}$ represents the vectorized form of the oblique parallelepiped $F_{m,n}$. Note that the dimensions of the matrix $H_{m,n}$ and the vector $f_{m,n}$ actually depend on the block size and the position of the windowed block in the measurement shots. Again, the equation above fits the standard CS formulation such that the matrix $H_{m,n}$ plays the role of the projection matrix and $f_{m,n}$ is the data vector, which is sparse in a certain domain. Equivalently,

(18) $$y_{m,n}=H_{m,n}\Psi_{m,n}^{T}x_{m,n}=A_{m,n}x_{m,n},$$

where $\Psi_{m,n}$ is the basis matrix with the appropriate size and $x_{m,n}$ is the sparse representation of $f_{m,n}$ in the domain defined by the considered basis.

Considering this block formulation, the main issue is now the construction of the set of measurement matrices $A_{m,n}$. In particular, the $H_{m,n}$ submatrices cannot be generated directly by partitioning the matrix H of the general model, but they have to be determined by considering the dispersion effect at a smaller scale. However, it is worth remarking that the effect of the $H_{m,n}$ submatrices over each block altogether must be equivalent to the use of the H matrix over the overall datacube.

**Figure 5:**
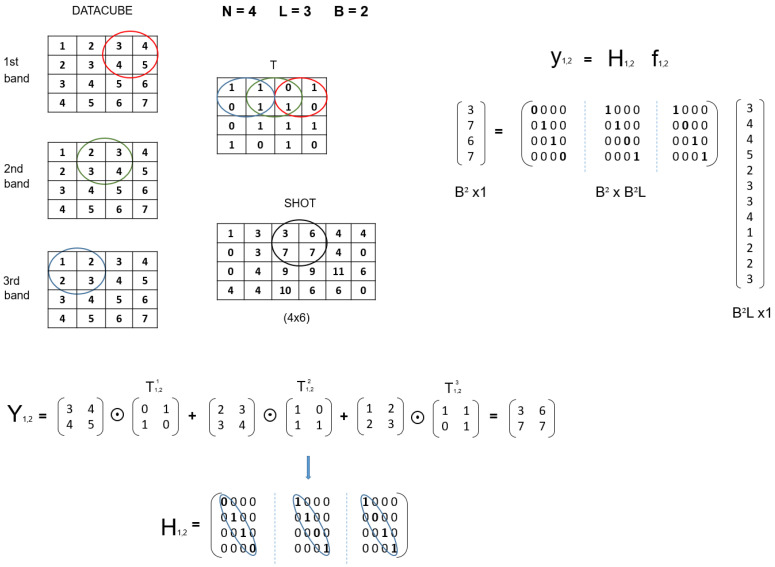
Example to illustrate the construction of the matrices $H_{m,n}$ in the block CASSI model.

Figure 5 shows a comprehensive example to illustrate the construction of matrices $H_{m,n}$ in the block CASSI model. As observed, the circled elements in the datacube slices represent the oblique parallelepiped through the L=3 spectral bands when considering a block size B=2. For the construction of the partial $H_{m,n}$ matrices, we first have to determine the section of the matrix T responsible for encoding the data of the considered parallelepiped at each slice. The same colors are employed to mark the involved sections in the matrix T for convenience. In the center of the figure, we show the result of applying the appropriate sections of the matrix T to each spectral band in the considered parallelepiped, which eventually produces the corresponding windowed 2×2 block from the whole measurement shot (in the general model). Finally, at the bottom of the figure, the construction of the appropriate partial H matrix for the considered windowed block is presented by assuming the required vectorization in the CS block formulation (see Equation (17)). As observed, it basically consists of vectorizing the involved sections of the matrix T, constructing diagonal matrices with the resulting vectors, and stacking them horizontally.

Considering the explained block model, we decomposed the general CS estimation problem for the CASSI model into a subset of problems with an equivalent formulation, but much smaller dimensions. A sparse estimation algorithm can hence be applied to each problem in order to obtain ${\hat{x}}_{m,n}$, which will correspond to the estimated vector obtained for the current block. As the last step, we have to perform an assembly procedure, which includes the following actions:

- For each block, we transform the estimate sparse vector ${\hat{x}}_{m,n}$ back to the original domain, obtaining ${\hat{f}}_{m,n}$;
- We reconstruct the corresponding oblique parallelepiped from ${\hat{f}}_{m,n}$;
- We place the parallelepiped at the appropriate coordinates within the 3D datacube.

As a result, we obtained a reconstruction of the input hyperspectral image given by the assembled datacube.

## 5. Experimental Results

In this section, we analyze the impact of the different factors involved in the reconstruction of hyperspectral images sampled with the CASSI setup. For that, we present the numerical results obtained from several computer experiments carried out to evaluate the practical suitability of the CASSI-based sensing procedure for hyperspectral images. In particular, the computer experiments comprise the following steps:

- For each input hyperspectral image, $N_{s}$ measurements shots were generated by using different random aperture patterns;
- The block CASSI model is defined for a particular value of the block size *B*. The partial measurement matrices $A_{m,n}$ were constructed for a given sparsifying basis and considering the aperture patterns to generate the shots;
- The nonoverlapping blocks of the obtained shots together with the corresponding partial measurement matrices were employed to reconstruct each oblique parallelepiped in the datacube. Different algorithms proposed for the sparse estimation problem were considered and assessed in this scenario;
- The datacube partial reconstructions were assembled to complete the recovering of the hyperspectral image from its compressive measurements;
- This procedure was repeated for all the items of an image database and the parameters of interest were averaged to obtain the numerical results presented in this section.

On the one hand, we employed a database containing 32 different images, which were specifically selected from several repositories for hyperspectral images [51,52,53]. The selection was performed with the aim of considering a wide variety of images with different features and levels of detail. These images included nature scenes, buildings and urban areas, people, different types of objects, etc.

On the other hand, since the main objective of this work was the evaluation of the CASSI sensing procedure for the sampling of hyperspectral images with an affordable computational cost, we were interested in analyzing the impact of different parameters in the block CASSI model: the number of shots $N_{s}$, the block size *B*, the type of sparsifying bases Ψ, and the sparse estimation algorithms. We considered two different relevant metrics to measure such an impact: the objective quality of the reconstructed images according to the PSNR metric and the computational cost according to the execution time required for the image reconstruction. Finally, an additional and indispensable criterion to assess the suitability of the different parameters is the visual or perceptual quality of the reconstructed images. All the computer simulations were conducted and timed using an Intel Core i7 4770 3.4 GHz processor and 32 GB RAM memory.

### 5.1. Sparsity Level

Before starting with the performance analysis of the block CASSI model, an interesting aspect to consider is the level of sparsity of the input hyperspectral images in the transformed domain depending on the chosen basis matrix. Towards this aim, we introduced the ratio between the number of zero coefficients and the total number of coefficients in the sparse domain, that is,

$$\gamma =\frac{\#\mathrm{zero}\mathrm{coefficients}}{\#\mathrm{total}\mathrm{coefficients}}\;,\;\;\;\mathrm{where}\;\gamma \in \left[0,1\right].$$

This figure of merit provides a measure of how much data can be compressed by a certain basis in the sparse domain. Indeed, large values of γ imply that the original data are sparser in the transformed domain or, equivalently, the considered transform is able to compact the signal energy into a smaller number of relevant coefficients.

Table 1 shows the sparsity level results obtained for different types of sparsifying bases and block sizes. The following conclusions were drawn:

- A clear conclusion is that DCT was the basis that obtained the sparsest representation of the data when it was transformed to the sparse domain. This is reasonable because wavelet-based transforms are known to work better when they are applied to the whole image (i.e., considerably large block sizes) to efficiently exploit the properties of the multilevel decomposition [54];
- For a similar reason, the simple Haar transform (single-level) provided poor sparsity values as it was only applied once to the different datacube blocks. Recall that the capacity of the energy compaction of wavelet transforms increases with the number of applied levels. This also explains that the sparsity level is practically the same for this basis regardless of the block size;
- DCT worked well even for small blocks as it approached the optimal Karhunen–Loève transform when the spatial information was highly correlated [48], as it was in this case;
- Increasing the block size in general increased the sparsity level. This conclusion matches the initial intuition. On the one hand, larger blocks usually led to larger data correlation levels for hyperspectral images. This fact benefits DCT-based schemes, which are able to represent more spatial information with a small increase in the amount of relevant frequency coefficients. On the other hand, larger blocks allow the utilization of multilevel wavelets with higher levels of decomposition;
- The increase in the sparsity level was especially remarkable for the Daubechies wavelet with four coefficients (DB4). Although DB4 provided lower levels of sparsity for small block sizes, its levels improve significantly when the block size increased, thus achieving a performance better than the multilevel Haar basis for blocks of size 32×32. Note that the basis matrix for DB4 had larger dimensions than for multilevel Haar because the number of wavelet coefficients was larger. This fact determines the maximum number of decomposition levels that can be applied to each datacube for a given size. As an example, for a block size 8×8, each datacube can be transformed using a three-level decomposition with multilevel Haar, whereas a two-level decomposition should be employed for DB4.

### 5.2. Objective Quality of the Reconstructed Images

In this subsection, we present the results obtained with different configurations of the CASSI setup in terms of the quality of the reconstructed images

#### 5.2.1. Impact of the Reconstruction Algorithm

**Figure 6:**
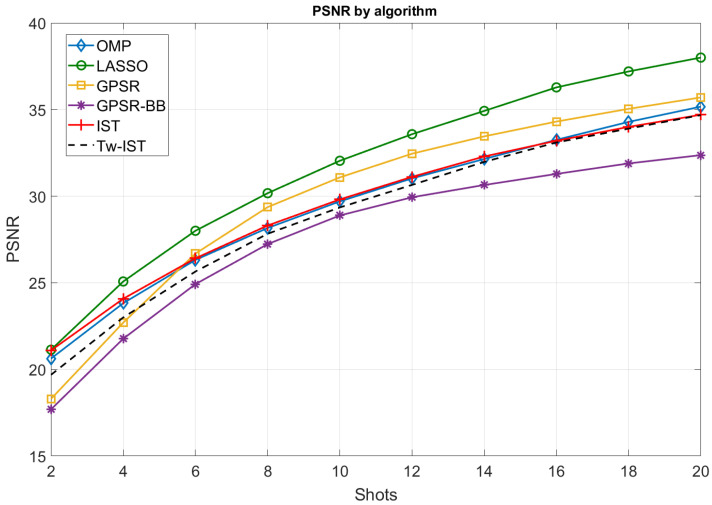
PSNR obtained for different sparse reconstruction algorithms.

In the first experiment, we measured the objective quality of the reconstructions for the considered sparse estimation algorithms by using the PSNR as a performance metric. Figure 6 shows the PSNR values achieved for different numbers of shots ($N_{s}$ from 2–20) and considering the following configuration for the CASSI setup: block size of 4×4 and DCT as the sparsifying basis. From these results, we highlight the following conclusions:

- As expected, the quality of the reconstructed images improved with the number of shots, i.e., when increasing the number of compressive measurements generated from the input image. The obtained PSNR ranged from low values (about 20 dB) for $N_{s}=2$ shots to excellent values (almost 40 dB) for a large number of measurement shots. As observed in Figure 6, the PSNR curves present a logarithmic behavior regardless of the sparse estimation algorithm. This means that the cost of incorporating more measurements is not worth it beyond a certain point;
- LASSO provided the highest average PSNR values regardless of the number of measurement shots. This is probably related to the fact that this algorithm is more robust than other alternatives such as OMP or IST when the columns of the measurement matrix are not completely orthogonal to each other. In our setup, such columns were not totally random because of the particular structure of the dictionary matrices H used for the projection operations (see Figure 5);
- OMP and IST recovered the hyperspectral images with similar quality. This makes sense as they intrinsically performed in a similar way even though the implementation of the operations at each iteration significantly differed;
- GPSR-based approaches provided the worst results when using a small number of shots. However, GPSR (basic implementation) was able to slightly outperform OMP and IST algorithms for $N_{s}\geq 6$ shots. This is an interesting observation as this class of algorithms stands out because of their lower computational cost, and hence, it was postulated as a low-complexity alternative to obtain accurate reconstructions, especially when a large number of measurements are available;
- The variant of IST based on using the Tw-IST algorithm converged to the performance obtained with the basic IST as the number of shots increased, but the quality of the reconstructions was slightly worse for a small number of measurements;
- Finally, we observed that GPSR-BB was clearly the worst algorithm in our setup. In this case, the alternative step initialization proposed by this method and the fact of allowing the cost function to increase along the iterative procedure did not apparently improve the performance for the CASSI reconstruction problem.

It is worth remarking that increasing the number of shots beyond 20 could be beneficial for most algorithms, as small gains still seem achievable. However, the fact of using more measurement shots led to storing/transmitting a larger number of samples (thus reducing the compression efficiency) and to a higher execution time required for the reconstruction of the images, as we see in Section 5.3. For this reason, it it essential to establish a trade-off between the benefits of image quality and the resource requirements.

As discussed, the improvement in the PSNR values followed a logarithmic tendency, and it apparently saturated for a large number of shots. This behavior agrees with the CS theory and with the sampling one, in general. According to previous works [17,18], the number of required measurements to obtain accurate approximations of the input data depends on the sparsity level and the properties of the measurement matrix (dictionary incoherence, mutual coherence between basis and projection matrices, etc.). From that point, the impact of increasing the number of measurements on the estimate accuracy is negligible and the images are reconstructed with a similar PSNR. In Figure 6, the curves show this saturation effect from the values of $N_{s}$ around 18 or 20 shots. These values approximately fit the well-known formula in the CS field to determine the number of required measurements assuming random projections [16], i.e.,

(19) $$M\approx C\;k\log_{2}\left(N\right),$$

where *M* is the number of measurements, whereas *k* and *N* are the number of nonzero elements and the total number of elements in the input vector, respectively. The letter *C* represents a small constant term.

#### 5.2.2. Impact of the Sparsifying Basis

**Figure 7:**
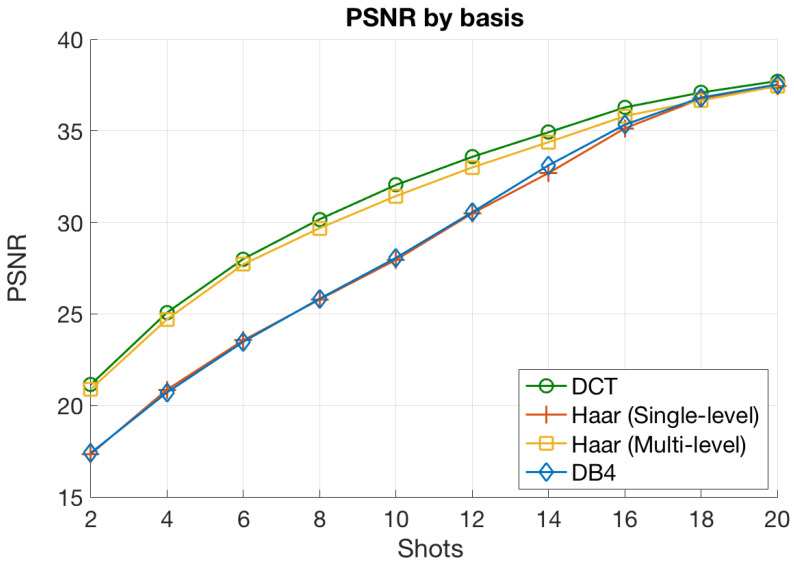
PSNR vs. the number of shots for different sparsifying bases.

We now consider a numerical experiment that focused on analyzing the impact of the basis used to perform the domain transformation. Figure 7 shows the average quality of the reconstructed images in terms of the observed PSNR values for the different bases considered throughout this work. The results were obtained for a CASSI setup with a block size of 4×4, LASSO as the estimation algorithm, and different numbers of shots. The decision of using LASSO was because it generally provided the best results, as we showed in the previous section.

When analyzing the results in Figure 7, we arrived at the following interesting conclusions:

- The DCT basis provided the highest PSNR values for all the range of $N_{s}$ values. Next, the multilevel Haar curve closely approached the DCT one, although the performance was slightly worse. Finally, the other two transforms (single-level Haar and DB4) achieved lower PSNR values, especially for a small number of shots. Notice that this behavior is coherent with the sparsity levels achieved by the set of bases for blocks of size 4×4 (see Table 1). In this setup, the DCT achieved the highest sparsity levels followed closely by the multilevel Haar, and further away, the other two transforms. A higher sparsity level directly implies a smaller number of nonzero coefficients of the input images in the sparse domain, and consequently, a better reconstruction with the available measurements;
- The PSNR curves for the four considered bases converged to the same point for a large number of measurement shots. This is also reasonable because the impact of the sparsity level vanished when the number of available measurement was enough to ensure reconstruction with minimum error, i.e., when that number was larger than the threshold provided by (19). As discussed, this situation only holds for $N_{s}$ values around 18 or 20. In such a scenario, the number of available measurements allowed accurate reconstructions for the four transform bases, even for those that produced vectors with a larger number of nonzero elements. Indeed, the improvement of obtaining sparser vectors in this situation was negligible in terms of the objective quality of the reconstructed images since the extra measurements hardly improved the accuracy in the estimation procedure.

According to these results and considering the sparsity levels in Table 1, the DCT basis seems to be a reasonable choice regardless of the rest of the parameters in the CASSI setup.

#### 5.2.3. Impact of the Block Size

In the next experiment, we were interested in evaluating the impact of the block size on the quality of the images reconstructed with the block CASSI model. We considered a CASSI setup configuration where the LASSO algorithm was employed at the estimation phase and the DCT was chosen as the sparsifying basis, since both elements were shown to provide the highest PSNR values in the previous experiments. We additionally considered the DB4 basis because it was expected to provide remarkable gains with the increase of the block sizes, as discussed in Section 5.1. Furthermore, we focused on a practical number of shots because of the prohibitive computational complexity and resource requirements when we combined large block sizes and a large number of shots.

**Figure 8:**
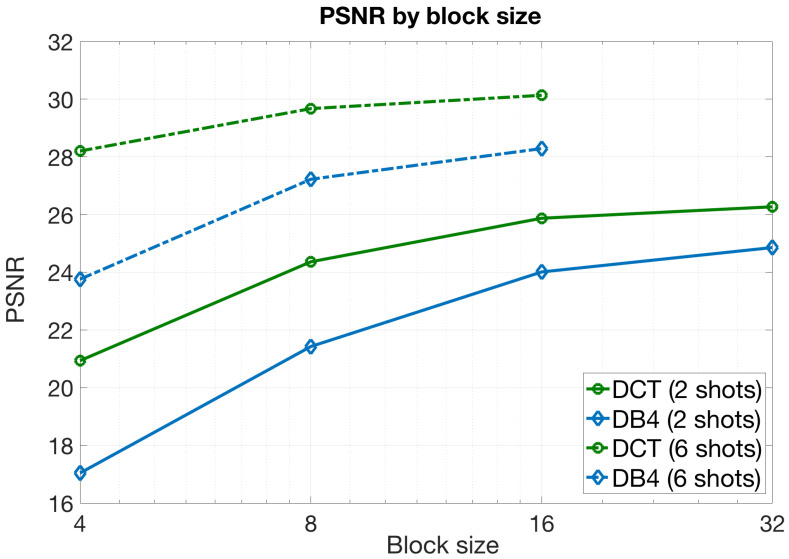
PSNR values by considering 2 and 6 shots, with different block sizes and using the DCT and DB4 basis.

Figure 8 shows the PSNR values obtained with the aforementioned configuration for different block sizes and for two different numbers of measurements shots, $N_{s}=2$ and $N_{s}=6$. In this case, we concluded the following:

- As expected, the PSNR values grew with the block size. Note that increasing the block size led to sparser vectors in the transformed domain, which could be estimated accurately. For each block, we can define the following quantities:
  $$\begin{array}{cc} \rho & =\left(1-\gamma \right)B^{2}L,\\ \xi & =B^{2}N_{s}, \end{array}$$
  where γ is the sparsity level, ρ is the average number of nonzero elements in the transformed domain at each block, and ξ is the number of measurements for each B×B block with $N_{s}$ shots. As observed, the relationship between these two variables did not depend on the block size *B* since both quadratically grew with the value of *B*. Thus, for fixed $N_{s}$ and *L* values in our CASSI setup, this relation was only determined by the sparsity level γ. In this sense, larger values of γ imply a smaller number of nonzero elements, and hence, we would be able to obtain more accurate reconstructions for each block with the same number of measurements. As observed in Table 1, the sparsity level generally grew with the block size, which justified the obtained results;
- The PSNR curves followed again a logarithmic tendency. Indeed, the gain in terms of PSNR was almost negligible from B=16 to B=32 for $N_{s}$ = 2 shots, and especially for the DCT. In the same way, the gain was imperceptible from B=8 to B=16 for $N_{s}=6$ shots. These results can be explained directly by the statement developed in the previous point and considering the increase of the sparsity levels with the block size presented in Table 1. As observed in the table, the sparsity level grew with the block size, but this increase slowed down as *B* became larger. This effect was even more pronounced when having more shots as we had a larger amount of measurements available for the reconstruction phase;
- The gains with the DB4 basis were slightly higher than with the DCT. Indeed, the curves tended to approach each other as *B* grew. This is reasonable as the Daubechies wavelet was shown to work better with larger data blocks, and it also agreed with the sparsity levels observed for this basis in Table 1.

A clear conclusion is that the benefits of using large block sizes were negligible when compared to the increase of the computational resources required to work with such sizes. A more practical approach consists of using small blocks with a large number of shots. This provides image reconstructions with higher quality and requires a lower consumption of resources.

### 5.3. Execution Time

In this subsection, we present and discuss the different results observed in the experiments concerning the execution time required to obtain the image reconstructions. Therefore, we exclusively measured the execution time employed to reconstruct each image from the considered data base and determined the average reconstruction time for each configuration of the block CASSI model.

#### 5.3.1. Impact of the Reconstruction Algorithm

In this experiment, we analyzed the execution time of the different considered algorithmic solutions: OMP, LASSO, IST, Tw-IST, and the two variants of GPSR. It is worth mentioning that the tuning of the parameters in the different reconstruction methods was performed with the aim of achieving the best possible objective quality. Therefore, the computational speed could be reduced in some scenarios at the expense of a lower reconstruction quality.

**Figure 9:**
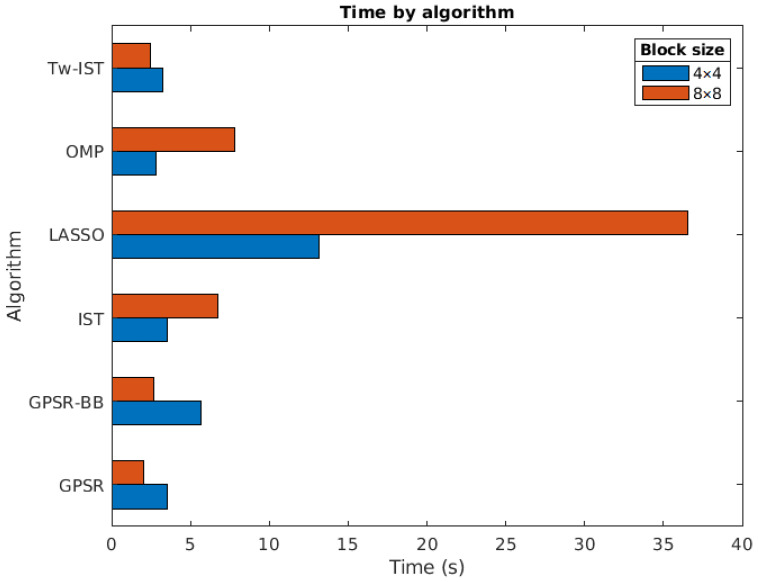
Execution time for the image reconstruction with different algorithms and block sizes.

Figure 9 shows the results obtained for all the sparse estimation algorithms considering 4×4 and 8×8 blocks and $N_{s}=2$ measurement shots. In this case, the DCT was used because the impact of the transform basis on the execution time was negligible. We next summarize the conclusions drawn from this experiment:

- The slowest method was, with a significant gap, LASSO. This is because of the number of iterations required for this method when the final goal is to achieve good quality results for the reconstructions. As already discussed in Section 3.1, this result was expected as LASSO usually requires a number of iterations larger than OMP or IST to converge to the final solution due to its strategy of handling the active set of candidates;
- OMP and IST had comparable times for both block sizes. This was due to the fact that, in both cases, the number of required iterations was closely related to the sparsity level of the solutions;
- The use of Tw-IST reduced the time required to reconstruct the hyperspectral images with respect to the basic version of IST, especially for 8×8 blocks. As observed, the obtained reconstruction times were of the same order as those of the GPSR-based methods;
- Both GPSR approaches were fast algorithms, with GPSR-Basic being the quickest one in both cases. They were clearly the fastest approaches for 8×8 blocks. Recall that the computational complexity of gradient-based algorithms is considerable lower than other iterative approaches based on pursuit methods or homotopy-based techniques [23];
- The execution time increased with the block size for OMP, IST, and LASSO. These three algorithms are iterative procedures where the number of iterations grows with the number of nonzero elements in the input vector to be estimated. It is clear that such a number would be larger for bigger input blocks as the total number of input elements quadratically grew with *B*;
- Conversely, the execution time for GPSR-based approaches was shorter for 8×8 than for 4×4. These results may seem counterintuitive, but the explanation of this behavior is closely related to the values of the regularization parameter τ obtained for each block size. In the case of 8×8, these values were in general lower than for 4×4, which provided less sparse solutions in the estimation procedure. In this case, the convergence of the gradient-based approaches was faster and the number of required iterations to produce the sparse solutions was smaller.

In summary, the LASSO algorithm provided the image reconstructions with the highest quality, but at the expense of larger execution times. These times also increased significantly with the block size and could become prohibitive for big blocks. An appealing alternative is the use of GPSR-Basic as it balances the reconstruction quality and the execution time.

#### 5.3.2. Impact of the Block Size

**Figure 10:**
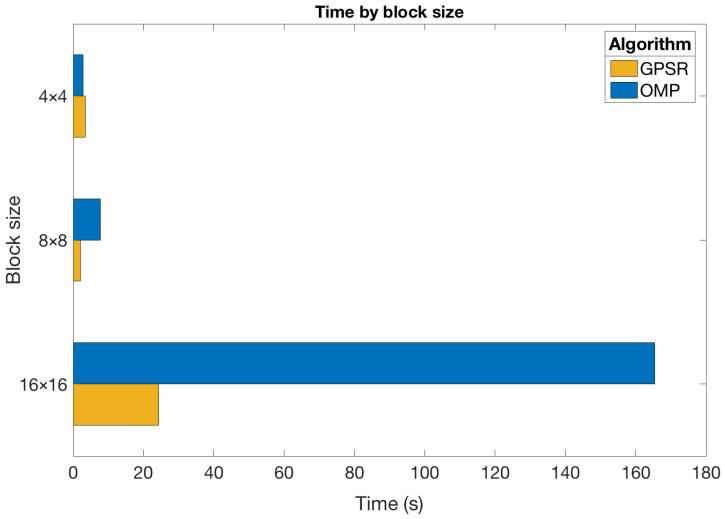
Execution time with different block sizes.

We now analyze the impact of the block size on the execution times required in the reconstruction step. In particular, we focus on a scenario where the DCT and $N_{s}=2$ shots were employed. Figure 10 graphically shows the obtained results for the OMP and GPSR-Basic algorithms considering different block sizes. These algorithms were chosen because they represent two seemingly fast and very different approaches, as shown previously. Therefore, it was of interest to verify how they behaved for different block sizes. In addition, Table 2 presents the execution times (in seconds) obtained for all the sparse estimation algorithms and the considered block sizes in this scenario.

Based on the results obtained, it is worth mentioning a few points:

- The execution time exponentially grew with the block size. Indeed, this time becomes prohibitive for most algorithms with 32×32 blocks. The only exception to this behavior is for GPSR-based approaches and the Tw-IST algorithm with 4×4 and 8×8 blocks, which was mentioned in the previous section;
- Execution times increased for the GPSR-Basic, GPSR-BB, and Tw-IST algorithms for sizes larger than 8×8. In this case, the dimension of the variables involved in the reconstruction problem significantly increased with B≥8, while the observed τ values were of the same order, which led to similar convergence properties. In any case, these approaches were still the fastest algorithms to reconstruct the hyperspectral images regardless of the block size;
- LASSO was clearly the slowest algorithm, and its execution time increase with the block size was more noticeable compared to the rest of algorithms. Indeed, the time for 16×16 blocks was already extremely high considering that it was even superior to the time of most algorithms for 32×32 blocks;
- OMP and IST required intermediate times to reconstruct the hyperspectral images. Nevertheless, the time growth was more pronounced in the case of the OMP algorithm. This indicates that IST could be more appropriate for large dimensions of the input vectors;
- Finally, these results confirmed that the use of Tw-IST significantly reduced the reconstruction times (by approximately half) with respect to the basic version of IST, especially as the block size was larger. As in the previous experiments, the execution times obtained with Tw-IST were slightly higher than for GPSR-based methods, but in the same order. These results suggest that the estimation problem with CASSI was not well-conditioned, probably due to the fact that the structure of the measurements matrix specifically depends on the considered aperture patterns, and hence, the resulting dictionary was not utterly incoherent.

Taking into account these points, we concluded that the slight quality gains obtained by increasing the block size did not actually compensate because of the huge increase in the involved resources, especially from the point of view of the execution times. This issue was less dramatic when using gradient-based approaches, so they represent an attractive alternatives under certain situations, as we discuss in Section 6.

### 5.4. Impact of the Number of Shots

In this last experiment, we evaluated the impact of the number of shots used in the reconstruction on the average execution time. For that, we considered a setup with the DCT as the sparsifying basis since it provided the best performance and the impact of using different bases on reconstruction times should be residual. We also focused on 4×4 and 8×8 blocks as in these cases, we can obtain reconstructions for a wider range of the number of shots with an affordable computational cost.

**Figure 11:**
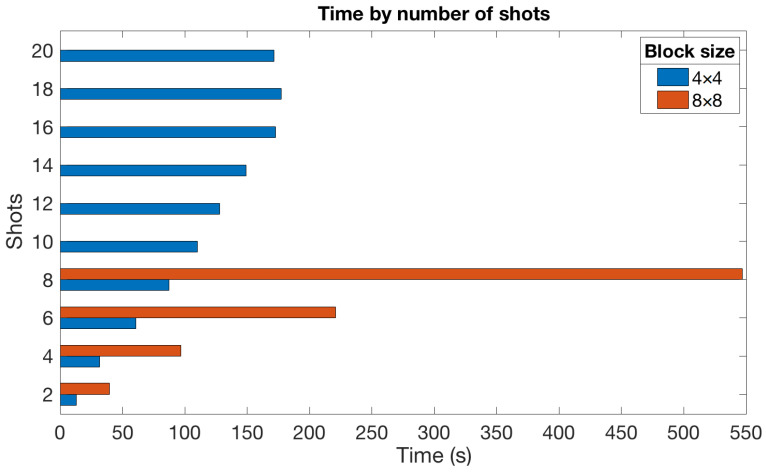
Execution time with different number of measurement shots and block sizes.

Figure 11 graphically shows the obtained results for the LASSO algorithm, whereas Table 3 provides the execution times for the rest of the estimation algorithms and a block size of 4×4. From these results, we highlight the following:

- All algorithms increased the time taken to reconstruct the images as more shots were considered. This fact meets our expectations since adding more shots implies increasing the variable dimensions and, therefore, increasing the execution time. This growth was more perceptible for a small number of shots, and it apparently lessened as $N_{s}$ became larger;
- LASSO was the slowest algorithm by far regardless of the number of shots. This agrees with the results obtained in the rest of the experiments;
- Focusing on the LASSO algorithm, the impact of increasing the number of shots was significantly larger for 8×8 blocks (see Figure 11). This was expected since the dimension of the involved variables also depended on the block size *B*;
- The GPSR-based algorithms generally provided the fastest times and showed a slight increase in time with the number of shots. This is an interesting result from the point of view of saving computational resources since GPSR-Basic was the best alternative for scenarios where a large number of shots was available.

### 5.5. Perceptual Quality

In this section, we show some illustrative examples of the reconstructed images with different configurations of the CASSI system. The objective was to complete the previous analysis by also considering the perceptual quality metric.

**Figure 12:**
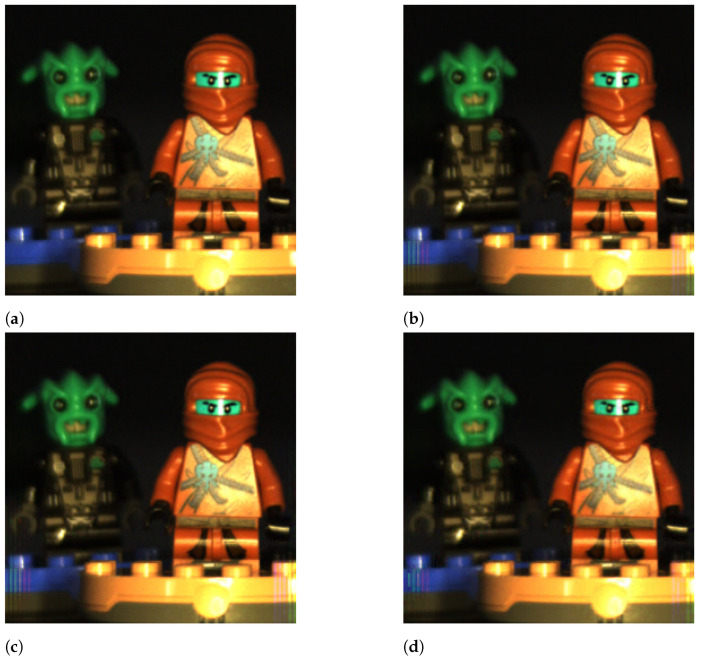
(**a**) Original 256 × 256 × 24 datacube for the “Ninja” image. Reconstructions for *N_s_* = 20 shots, DCT basis, and 4 × 4 blocks using: (**b**) the LASSO algorithm (PSNR = 37.41 dB), (**c**) the OMP algorithm (PSNR = 35.89 dB), and (**d**) the GPSR-Basic algorithm (PSNR = 36.24 dB).

In particular, we used a 256×256×24 hyperspectral image as the reference, which is shown in Figure 12a, by considering only the spectral bands corresponding to its RGB components. Figure 12 shows the original hyperspectral image and the obtained reconstructions with the LASSO, OMP, and GPSR-Basic algorithms for a configuration with $N_{s}=20$ shots, 4×4 blocks, and the DCT basis. As observed, the perceptual quality of the three reconstructed images was similar, and the differences with respect to the original one were negligible. The details and colors of the original image were also accurately represented in the three reconstructed images. Despite the fact that LASSO achieved over 1 dB more than GPSR and over 1.5 dB more than OMP, this gain is hardly perceptible if we compare the three reconstructed images.

**Figure 13:**
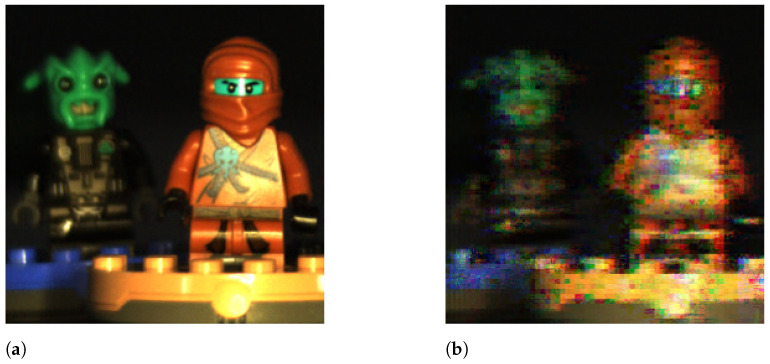

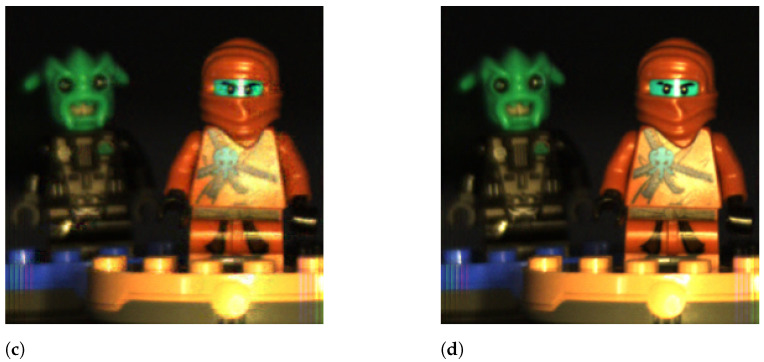
(**a**) Original 256×256×24 datacube for the “Ninja” image. Reconstructions using the LASSO algorithm, the DCT basis, and 4×4 blocks with: (**b**) 2 shots (PSNR = 19.42 dB), (**c**) 10 shots (PSNR = 30.47 dB), and (**d**) 20 shots (PSNR = 37.41 dB).

Figure 13 shows the original hyperspectral image and the obtained reconstructions for LASSO, 4×4 blocks, the DCT basis, and $N_{s}=$ 2, 10, and 20 shots. As observed, the number of shots in the reconstruction procedure decisively impacted the perceptual quality of the obtained reconstructions. With two shots, the ninja figures in the image are blurred and the edges of the different elements are not clearly defined. With 10 shots, the quality of the image significantly improved as the image details are sharp and the edges clearly defined. However, some artifacts are still visible at the bottom of the images in the Lego bricks, and some of the colors are a bit dull. With 20 shots, the reconstructed image is almost identical to the original one, and the high PSNR value obtained reflects this fact. These graphical results confirmed the weight of the number of shots in the image quality, higher than other variables, such as the sparse estimation algorithm.

**Figure 14:**
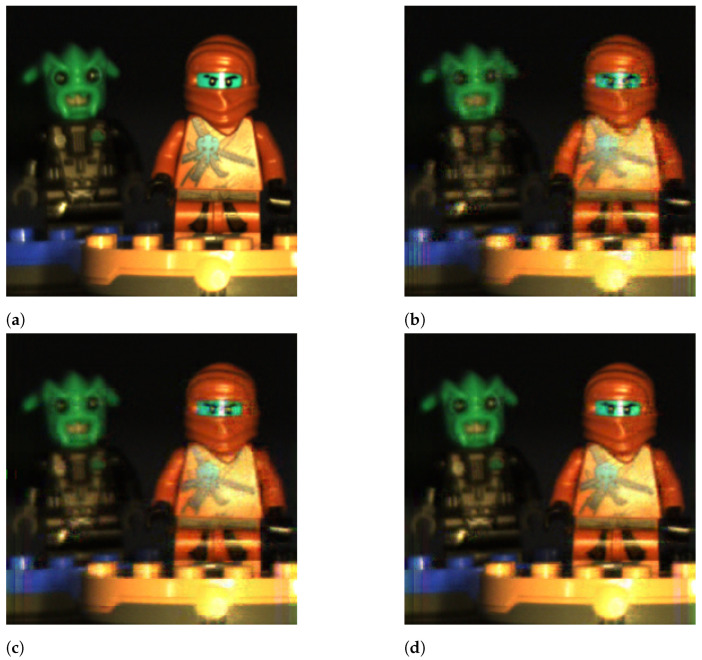
(**a**) Original 256 × 256 × 24 datacube for the “Ninja” image. Reconstructions using 6 shots, the DCT basis, and the LASSO algorithm and considering a block size of: (**b**) 4 × 4 (PSNR = 26.44 dB), (**c**) 8 × 8 (PSNR = 29.11 dB), and (**d**) 16 × 16 (PSNR = 30.57 dB).

Figure 14 shows the original hyperspectral image and the obtained reconstructions for LASSO, the DCT basis, $N_{s}=6$ shots, and B= 4, 8, and 16. As observed, the perceptual quality of the images improved as the blocks became bigger. This improvement was more noticeable when we increased the block size from 4×4 to 8×8, although it was also perceptible when increasing the size from 8×8 to 16×16. Indeed, the visual differences between the images in Figure 14c,d are higher than indicated by their PSNR values.

## 6. Discussion

The use of CASSI-based hardware prototypes for the sampling of hyperspectral images represents a promising strategy because it is able to exploit the optical properties of the light waves to reduce the amount of information that we need to capture from a given scene. In addition, the underlying CASSI principles can be modeled from the perspective of the CS theory, thus exploiting the vast mathematical fundamentals developed for it. It is obvious that this point should be seen as a positive point, although it also implies that there exists a wide variety of potential techniques that could be applied for the reconstruction of hyperspectral images from their compressive measurements in this setup. For this reason, it is interesting to analyze those reconstruction strategies and the involved parameters for the design of practical CASSI systems. In addition, the best configuration for the whole system, especially regarding the reconstruction phase, depends on multiple factors such as the application requirements or the computational resources of the devices.

The lack of specific works that analyze in detail such aspects for CASSI has motivated the study conducted in this paper. Considering the results of all the computer experiments presented along the previous section, we would like to highlight the following general conclusions:

- LASSO was the algorithm that provided the image reconstructions with the highest objective quality. It was able to provide excellent performance in spite of the particular structure of the columns in the CASSI measurement matrix. However, these better results were achieved at the expense of increasing the execution time required for the reconstruction step, especially when employing a larger number of shots or larger block sizes. Hence, LASSO is the best alternative for applications where it is essential to work with high-quality reconstructions, for applications without delay constraints, and for devices with enough computational capabilities;
- GPSR-Basic is an appealing alternative as it properly balanced image quality and time consumption. It was especially interesting for the case of many shots since, in this situation, it was able to reconstruct the hyperspectral images with excellent quality and negligible impact on the time consumption;
- The best option to improve the quality of the image reconstructions is to increase the number of shots generated from the input scene. If possible, this alternative is preferable to increasing the block size in the block CASSI model, both in terms of quality improvement and required reconstruction time. From the reconstruction perspective, the increase of the generated shots allows producing hyperspectral images with excellent quality at an affordable computational cost and delay. The use of larger block sizes for a given number of shots led to a slight improvement of the reconstruction quality at the expense of increasing the computational cost and reconstruction delay. However, generating more shots implies more hardware complexity, more measurements to be stored/transmitted, and lower compression efficiency. Again, the final decision about the system configuration will depend on the application requirements, the hardware features of the CASSI prototype, and the computational resources of the devices where the hyperspectral images will be reconstructed;
- In those scenarios where it is not possible to generate a large number of shots, we can opt to increase the block size. In any case, the decision of using LASSO or GPSR will depend on the available computational resources;
- The benefits of generating more shots for the hyperspectral image vanished beyond a certain point, which can be determined from the CS theory. This threshold value will mainly depend on the sparsity level of the input image;
- The impact of the sparsifying bases on the system performance is less relevant than that of other configuration parameters. In any way, the DCT basis is apparently the best choice for the considered block sizes as it is able to produce sparser vectors in the transformed domain.

This work presented a comprehensive study of the reconstruction of hyperspectral images sampled with CASSI techniques. However, there exist some points that can be addressed in the future:

- The use of more sophisticated and modern reconstruction algorithms, including those based on deep learning networks;
- The impact of the transmission of the measurements over a certain communication channel on the reconstruction procedure and, correspondingly, the impact of noisy measurements on the reconstruction of hyperspectral images;
- The optimization of the coded aperture patterns for the generation of the measurement shots;
- A comparison of the considered block model with respect to using overlapped blocks in the reconstruction problem.

## 7. Conclusions

In this work, we addressed the sensing and reconstruction of hyperspectral images. The spectral information from the scenes was assumed to be acquired and sampled with a CASSI-based device, which allowed modeling the sensing and subsequent reconstruction with the support of CS fundamentals. Considering a practical implementation of this model based on nonoverlapping blocks, we analyzed the impact of several parameters on the reconstruction procedure in terms of the quality of the recovered images and the time required for their reconstruction. This study provides an interesting insight for an appropriate design of the overall CASSI system depending on the application requirements and the available computational resources.

Several interesting conclusions can be extracted after completing this study. The preferable strategy to improve the quality of the reconstructions is the increase of the number of shots generated from the hyperspectral scene. This alternative can also be combined with the use of a low-complexity reconstruction algorithm such as GPSR to construct an overall practical CASSI-based system with an affordable consumption of hardware resources. If possible, this strategy allows producing hyperspectral images with excellent quality at an affordable computational cost and delay. However, there could be certain situations where this strategy cannot be applied due to hardware limitations in the CASSI camera, limited storage resources, or restrictions on the available bandwidth for transmission scenarios. In these situations, the imposition of using a small number of measurement shots tips the scale for the use of the LASSO algorithm in order to leverage the suitable performance of this approach even with a small amount of measurements from the input images.

This paper focused on analyzing some classical model-driven algorithms that are usually employed for the reconstruction of sparse data in the context of CS. It represents a preliminary approach to assess different alternatives to reconstruct hyperspectral images sampled with CASSI-based techniques. However, in the future, it would be interesting to incorporate more sophisticated algorithms in this comparison, especially those based on the use of deep learning techniques.

In a similar way, the reconstruction procedure considered in the paper was based on splitting the measurements shots into nonoverlapping blocks, applying the corresponding estimation algorithm separately to each block, and reassembling the datacube by considering the dispersive effect. In the literature, other strategies have been proposed to decompose the reconstruction procedure into small-scale subproblems for CASSI scenarios such as, for example, the use of overlapping blocks to improve the quality of the reconstructions. However, this idea implies changing the construction procedure for the measurement matrix and the way of reassembling the partial reconstructions into the three-dimensional datacube. In this sense, an interesting continuation of this work would be to follow a similar approach for the overlapped block model and compare the results to those achieved with the nonoverlapping model considered in this work.

## Figures and Tables

**Table 1:** Level of sparsity (γ) for different bases.

Basis/Block Size	4 × 4	8 × 8	16 × 16	32 × 32
DCT	0.8717	0.9030	0.9180	0.9296
Haar (single-level)	0.8052	0.8072	0.8097	0.8105
Haar (multilevel)	0.8564	0.8752	0.8852	0.8905
Daubechies (DB4)	0.8034	0.8582	0.8835	0.9057

**Table 2:** Execution time (in seconds) required for the image reconstruction with $N_{s}=2$ shots, the DCT basis, and considering different algorithms and block sizes.

Algorithm/Size	4 × 4	8 × 8	16 × 16	32 × 32
OMP	2.8510	7.7574	165.3975	1784.93
LASSO	13.1868	36.5139	524.9444	5940.48
GPSR	3.4153	2.0287	24.3466	188.75
GPSR-BB	5.6395	2.5284	29.8273	229.26
IST	3.4501	6.7514	70.8613	512.07
Tw-IST	3.2421	2.3771	31.0239	243.58

**Table 3:** Execution time (in seconds) required for the image reconstruction with B=4, DCT basis, and different algorithms and the number of shots.

Algorithm/#Shots	2	8	14	20
OMP	2.8510	25.0447	55.8019	70.2041
LASSO	13.1868	87.1824	143.0577	169.5150
GPSR	3.4153	5.9478	7.3869	7.9944
GPSR-BB	5.6395	6.9739	8.1231	8.6928
IST	3.4421	17.1846	30.2728	38.8165
Tw-IST	3.2421	6.1562	8.1849	9.3319

## Abbreviations

The following abbreviations are used in this manuscript:

AMP	Approximate Message-Passing
BCQP	Bound-Constrained Quadratic Programming
CASSI	Coded Aperture Snapshot Spectral Imagers
CS	Compressive Sensing
DCT	Discrete Cosine Transform
DWT	Discrete Wavelet Transform
FPA	Focal Plane Array
GPSR	Gradient Projection Sparse Reconstruction
IHT	Iterative Hard Thresholding
IoT	Internet of Things
IST	Iterative Shrinkage Thresholding
LASSO	Least Absolute Shrinkage and Selection Operator
OMP	Orthogonal Matching Pursuit
PSNR	Peak Signal-to-Noise Ratio
Tw-IST	Two-Step Iterative Shrinkage/Thresholding
